# Balancing Benefits and Risks of Indomethacin in the Management of Antenatal Bartter Syndrome: A Case Report

**DOI:** 10.3389/fmed.2022.870503

**Published:** 2022-06-29

**Authors:** Omar Ala' Alajjuri, Mayar Essam Samaha, Ulrich Honemeyer, Ghada Mohammed, Noha A. Mousa

**Affiliations:** ^1^College of Medicine, University of Sharjah, Sharjah, United Arab Emirates; ^2^New Medical Center (NMC) Royal Hospital Sharjah, Sharjah, United Arab Emirates; ^3^Clinical Sciences Department, College of Medicine, University of Sharjah, Sharjah, United Arab Emirates

**Keywords:** polyhydramnios, Bartter syndrome, indomethacin, Ductus Arteriosus, amniotic fluid index

## Abstract

**Background:**

Bartter syndrome, a very rare inherited renal tubular disorder, characterized by urinary salt wastage, hypokalemia, polyuria, and metabolic alkalosis, may manifest antenatally as severe isolated polyhydramnios. Indomethacin is known to reduce salt wastage and subsequent polyhydramnios during pregnancy; however, it reduces the Ductus Arteriosus diameter among other potential complications, such as inhibition of gastrointestinal perfusion and increasing the risk of renal toxicity.

**Case:**

A 36-year-old multigravida presented with severe isolated polyhydramnios at 30 weeks of gestation. Based on a history of a previous pregnancy affected with Bartter syndrome, indomethacin was initiated. Amniotic fluid volume and Ductus Arteriosus diameter were monitored. As evidence lacks on optimal dose and duration of indomethacin, multiple-dose adjustments were made to reduce the amniotic fluid volume while maintaining normal Ductus Arteriosus diameter. Progressive polyhydramnios led to Cesarean section at 34+ weeks of gestation resulting in a healthy fetus diagnosed with Bartter syndrome in the early neonatal period.

**Conclusion:**

We share our experience in the adjustment of the dose and duration of Indomethacin therapy in the treatment of severe polyhydramnios associated with antenatal Bartter syndrome. Amniotic fluid index, Ductus Arteriosus diameter, and umbilical artery doppler work together as key indicators to guide the success and safety of the therapy.

## Background

Antenatal Bartter syndrome (BS) is a rare inherited renal disorder, due to an autosomal recessive defect of several genes identifying five different types of BS, including *SLC12A1* on chromosome 15, *KCNJ1* on chromosome 11, *CLCNKA, CLCNKB*, and *BSND* on chromosome 1, while mutation of the *MAGED2* gene is X-linked. BS is mainly a renal tubular disorder characterized by hypochloremia and polyuria associated with a severe urinary loss of sodium chloride. Accordingly, hyperreninemia and hyperaldosteronism develop, which result in hypokalemia and metabolic alkalosis with a normal to low blood pressure. Moreover, prostaglandin E2 production is increased, which leads to electrolyte abnormalities by direct stimulation of the release of renin from the juxtaglomerular cells in the kidney (1–3). Most of these biochemical and metabolic abnormalities can be diagnosed in the neonatal or childhood period based on the severity of BS which affects the onset and extent of symptoms.

The most prominent ***antenatal*** clinical feature when the fetus is affected by BS is early-onset maternal polyhydramnios, starting at a median age of 19–20 weeks of gestation, as a result of fetal polyuria and salt wastage (4). Other reported ***antenatal*** complications of BS include intrauterine growth restriction, premature rupture of membranes, and preterm birth (2). In normal pregnancies, the amniotic fluid volume varies at different gestational weeks with a median amniotic fluid index (AFI) of around 14 cm from week 20 to 35, after which the amniotic fluid volume begins to decline to reduce. Generally, an AFI between 5 and 25 cm is considered within physiological limits (5). Polyhydramnios is accordingly defined by an AFI of more than 24–25 cm (95–97th centile) or a single deepest vertical pool (DVP) of more than 8 cm. The severity of polyhydramnios is further categorized as mild (AFI of 24.0–29.9 cm or DVP of 8–11 cm), moderate (AFI of 30.0–34.9 cm or DVP of 12–15 cm), and severe (AFI >35 cm or DVP >16 cm) (6, 7).

Currently, there are no clinical guidelines to support evidence-based management of BS during the antenatal period, and research data is very scarce. Antenatal administration of indomethacin to the mother has been reported to reduce fetal salt wastage and polyuria of BS, subsequently reducing polyhydramnios (8). However, indomethacin use is associated risks, most concerning during pregnancy is its effect on the patency of the Ductus Arteriosus (DA) (2, 9). Oral indomethacin has been prescribed for pregnant women for the treatment of polyhydramnios or for other indications (e.g., tocolysis in preterm labor) in doses that range between 1 and 3 mg/kg/day for variable durations of a few days to several weeks (2, 9, 10). However, there is no adequate data on its optimal dose or duration in the antenatal BS. In this report, we discuss challenges in using indomethacin for the management of antenatal BS. We share our experience in adjusting the dose and duration of indomethacin in the treatment of a patient who presented with severe polyhydramnios associated with antenatal BS. Clinical and sonographic indicators were combined to enhance the success and safety of the therapy for the mother and the fetus.

## Case Presentation

A 36-year-old female gravida five with a history of three miscarriages and a preterm twin delivery, presented at 21 weeks of gestation for a routine anomaly scan. Her obstetric history was remarkable for a previous dichorionic diamniotic twin pregnancy 5 years earlier during which the patient developed severe polyhydramnios in one of the twins, resulting in a Cesarean section at 34 weeks. Bartter syndrome was suspected due to the severe isolated polyhydramnios and the normal fetal and placental structures; however, no further diagnostic steps were taken. Confirmation of the diagnosis was made 3 weeks following delivery, based on elevated urinary sodium, potassium, and chloride. Hypokalemia, high plasma renin, and metabolic alkalosis were also detected. The affected female child survived and has been on sodium supplements and ibuprofen since birth up to the ages of 6 and 36 months, respectively. Her growth was initially slow, however, by the age of five, she has maintained normal physical and mental growth.

**Figure 1:**
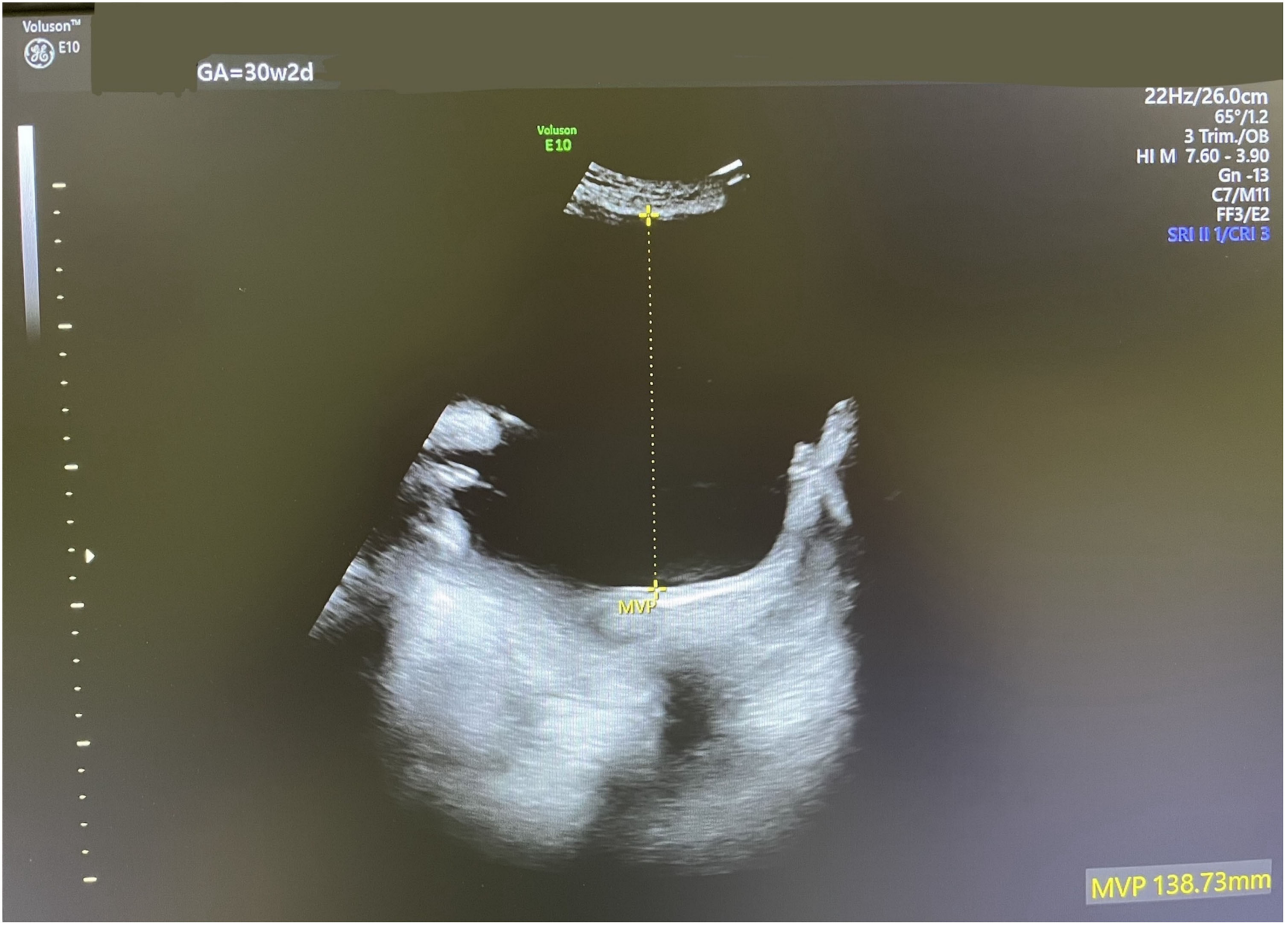
An ultrasound scan showing severe polyhydramnios with a single DVP of 13.9 cm at 30 weeks and 2 days.

In this reported pregnancy, a dating scan was performed at 12 weeks showing no gross fetal anomalies. The anomaly scan at 21 weeks showed a female fetus with no structural abnormalities. Amniotic fluid volume was normal with a single deepest vertical pocket (DVP) of 6 cm. Fetal biometry was consistent with the gestational age. At 30 weeks + 2 days, a follow-up ultrasound scan showed significant polyhydramnios with an AFI of 35 cm and a single DVP of 13.9 cm (Figure 1). Investigations to exclude other causes of polyhydramnios, including complete blood count, Parvovirus IgG, and IgM antibodies, and oral glucose tolerance test were all normal. Amniotic fluid genomic DNA analysis was declined by the patient due to financial restraints.

**Figure 2:**
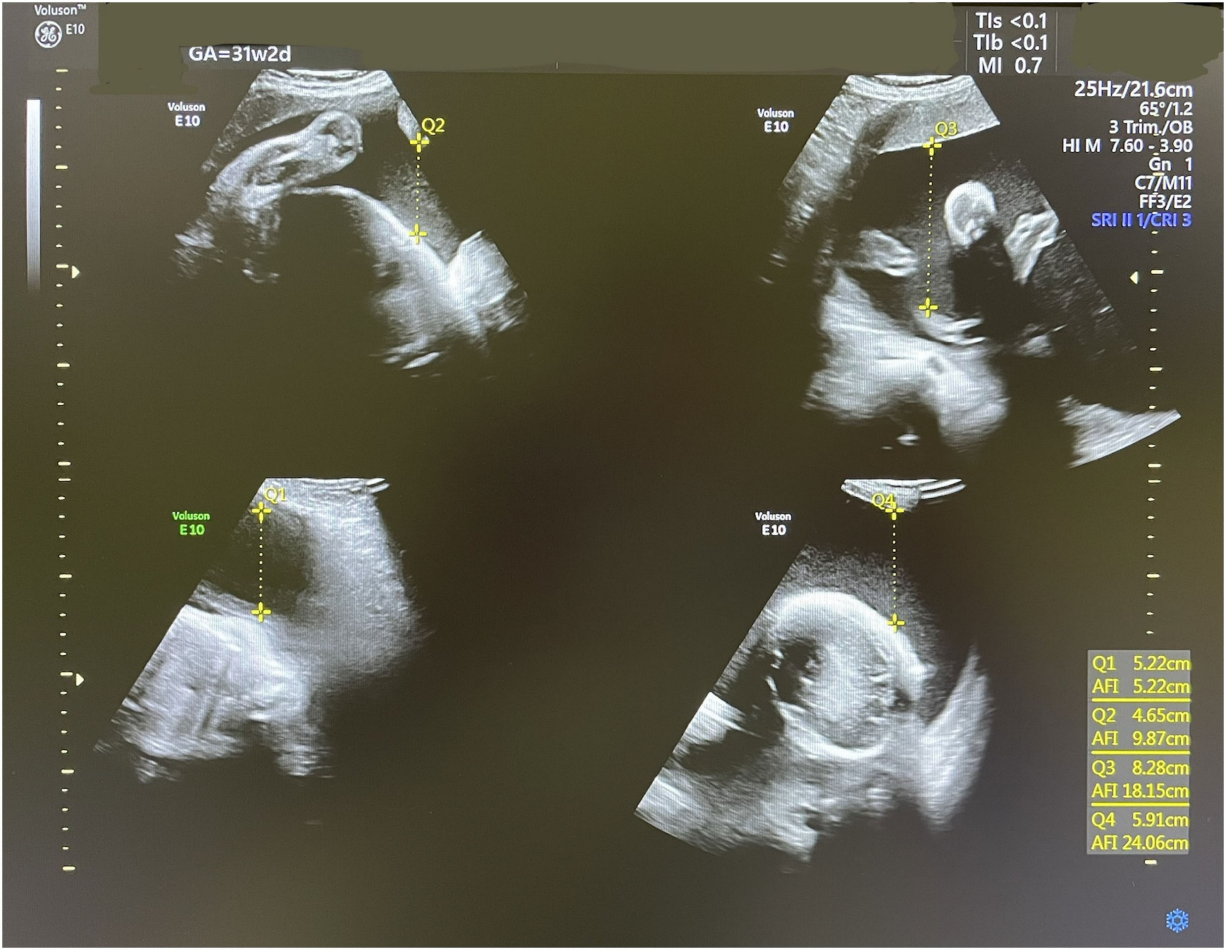
An ultrasound scan showing an AFI of 24.1 cm at 31 weeks 2 days.

**Figure 3:**
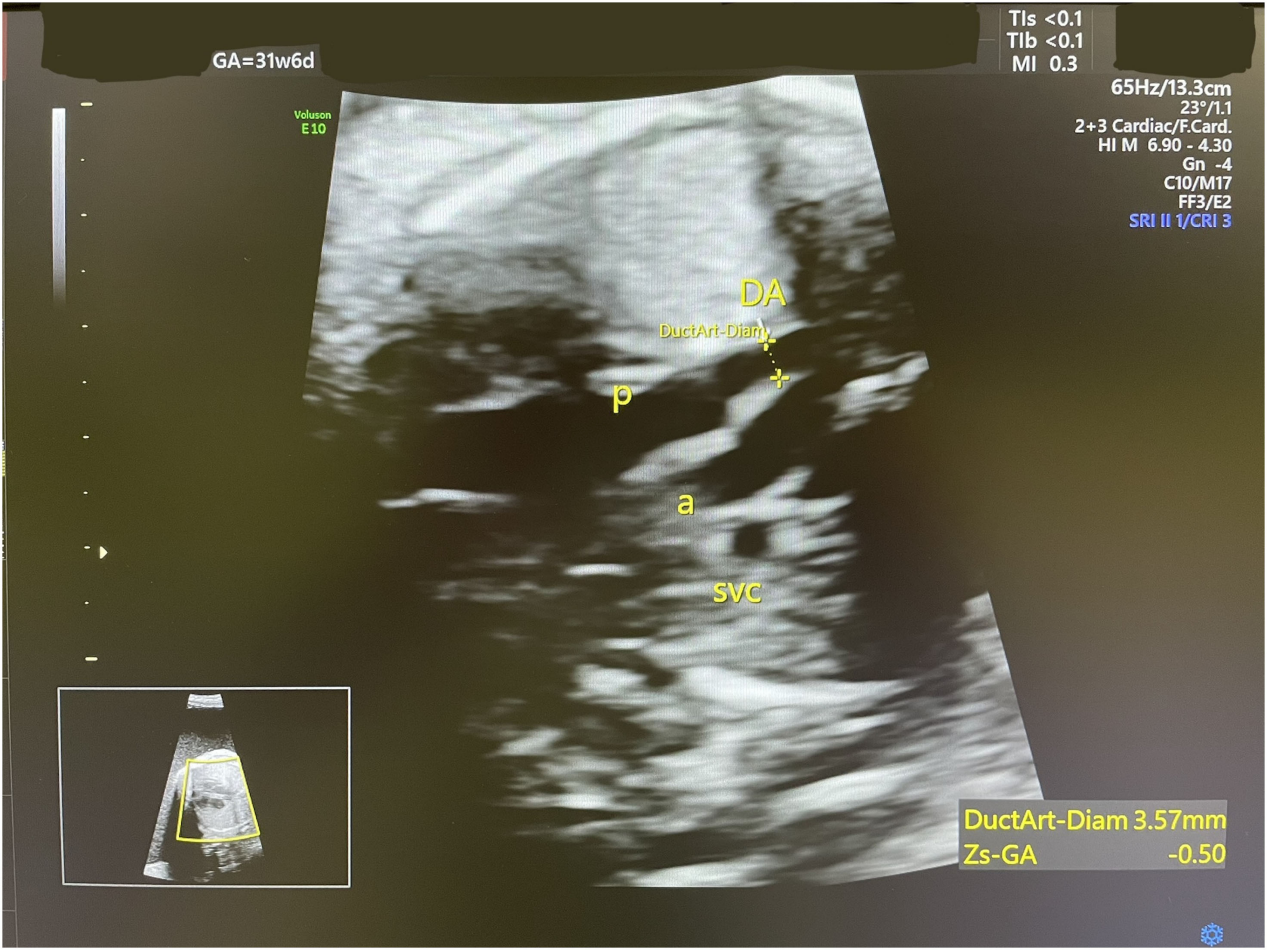
An ultrasound scan shows DA diameter at 31 weeks 6 days (3.57 mm, 6th centile).

Accordingly, indomethacin was started at 30 weeks + 2 days at a dose of 150 mg/day and the AFI and DA diameter were sonographically monitored. One week later, the AFI decreased from 35 to 24.1 cm (Figure 2), while the DA diameter significantly decreased from 3.8 to 3.1 mm (corresponding to the 1st centile). The dose of indomethacin was accordingly reduced to 50 mg/day. Four days later, the AFI increased to 28 cm, while the DA diameter increased to 3.57 mm [corresponding to the 6th centile (Figure 3)]. Since it is not recommended to use indomethacin after 32 weeks (11), it was tapered down over 2 days until it was stopped at 32 weeks + 2 days. At that time, the AFI reached 30 cm, whereas the DA diameter increased to 3.98 mm (corresponding to the 19th centile).

**Figure 4:**
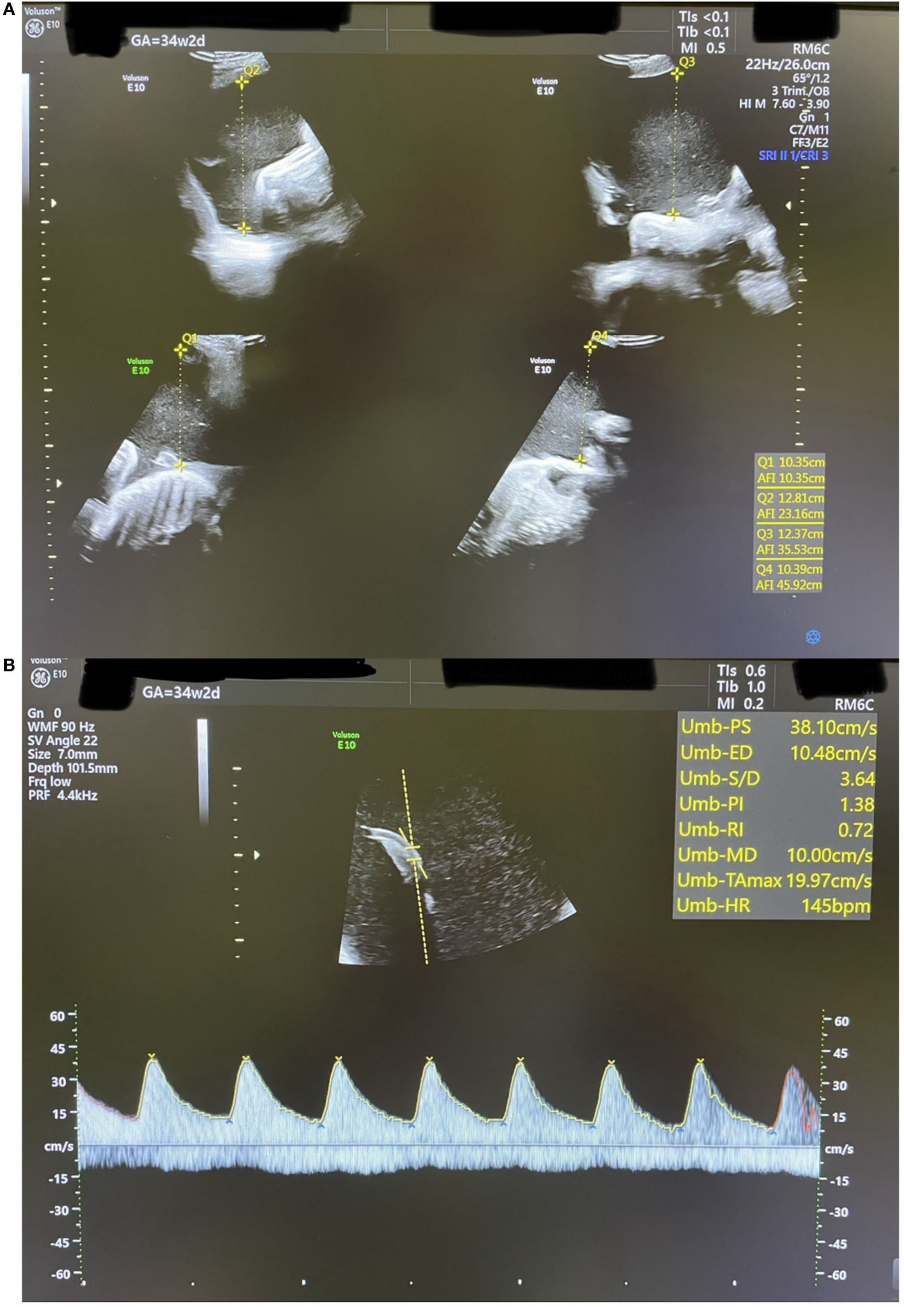
An ultrasound scan showing findings at 34 weeks + 2 days **(A)**: AFI of 45.9 cm. **(B)**: Umbilical artery Doppler waveform with increased PI above the 99th centile.

**Figure 5:**
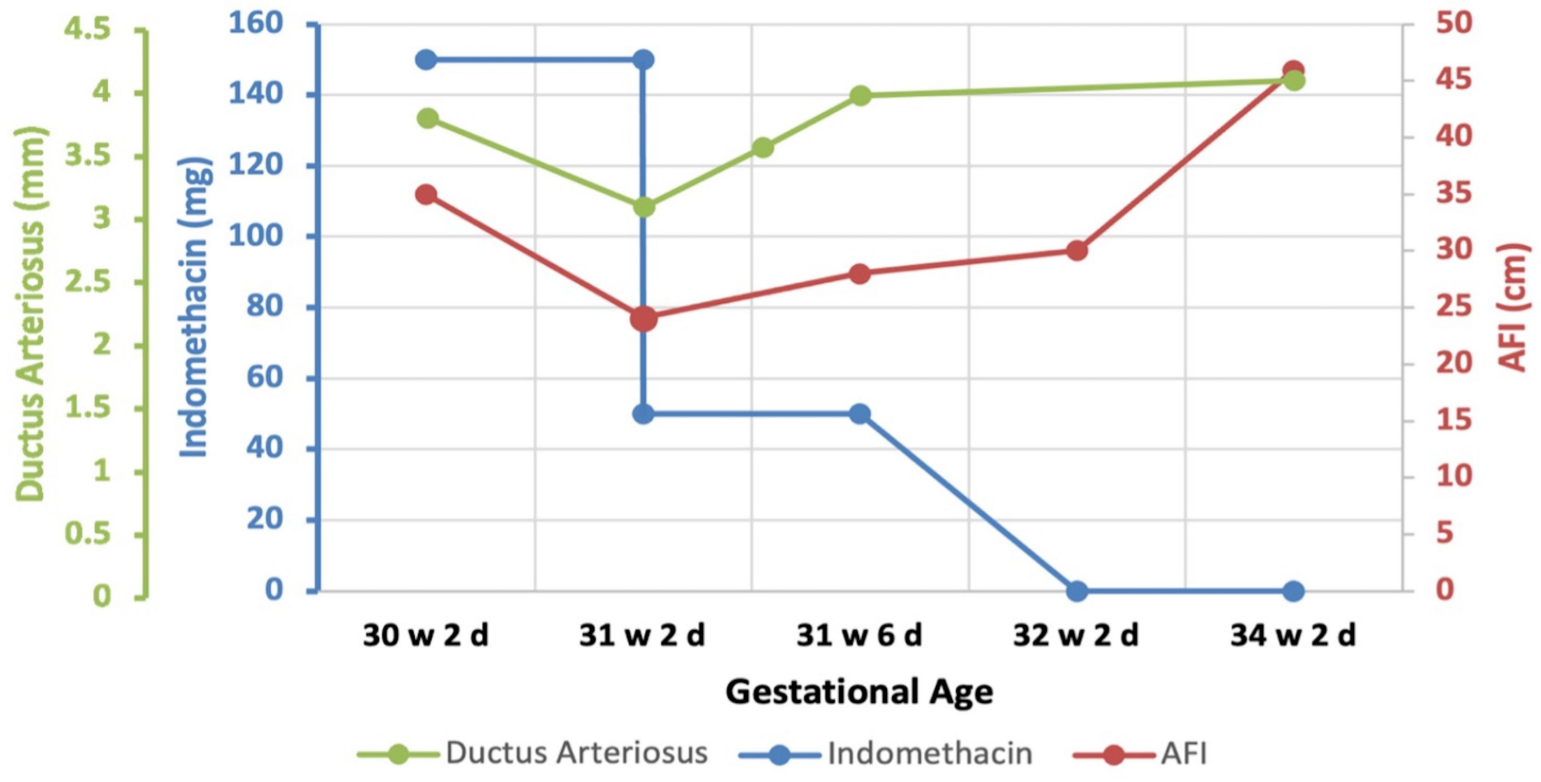
A summary diagram to outline changes in AFI and DA diameter during the antenatal indomethacin therapy of the patient.

Two weeks later, the patient started to be symptomatic with increasing abdominal pain and shortness of breath with a significant increase in AFI at 45.9 cm (Figure 4A). At that point, the umbilical artery pulsatility index (PI), which was previously normal, increased considerably to 1.38 (Reference range 0.60–1.28 at 34 weeks, i.e., above the 99th centile) (12) (Figure 4B). A summary of the changes in AFI and DA diameter in response to indomethacin dose adjustments is shown in Figure 5.

Accordingly, the decision to deliver the patient by a Cesarean section was made, noting that corticosteroids were prophylactically administered at 30 weeks. A healthy neonate was delivered at 34 weeks + 3 days with a birth weight of 1,615 grams and an APGAR score of 10/10 at 1 min. On the second day postpartum, the investigations showed elevated neonatal serum aldosterone at 12,500 ng/l (Reference range 30–90 ng/l), and serum renin at 255.7 ng/ml/h (Reference range 2–37 ng/ml/h). The initial urine output was 15 ml/kg/h (normal range 1–2 ml/kg/h) which decreased to 7 ml/kg/h after starting ibuprofen. Potassium chloride supplements were given up to 36 months of age until potassium levels were normalized. The infant developed necrotizing enterocolitis twice which were managed surgically. Ibuprofen was, therefore, stopped at the age of 3 months, and the parents refused to restart in fear of complication recurrence. At the time of writing this report, the child was 3.5 years old and continued to grow at a slow rate, likely due to discontinuation of the NSAID treatment or the effect of repeat small bowel resections. However, the infant's mental development and hearing function have been normal. The renal function has remained within normal limits.

## Discussion

Differential diagnoses of isolated polyhydramnios include gastrointestinal anomalies, aneuploidies, diabetes, anencephaly, neuromuscular conditions such as X-linked myotubular myopathy, or myotonic dystrophy, congenital high airway obstruction syndrome, and Bartter syndrome. In addition, other conditions associated with high fetal cardiac output can cause polyhydramnios such as large arteriovenous fistulas, fetal anemia, and fetal infections, mainly Parvovirus B19 (13). In the present case study, Bartter syndrome (BS) was highly suspected based on the history of a previously affected sibling (i.e., an index case) and the exclusion of other differentials. Without such suggestive history, the diagnosis of antenatal BS requires a high index of suspicion since it remains a rare antenatal condition (14). It should be therefore considered, after excluding more common causes of polyhydramnios.

Prenatal genomic DNA analysis from cultured amniotic cells is recognized as the most definitive diagnostic test for antenatal BS. In most cases, this will identify the affected gene and type of BS, each can have a different prognosis [For example, BS type 1 is caused by a mutation in the SLC12A1 gene, while BS type 2 is caused by a mutation in KCNJ1 gene (1, 15)]. Nonetheless, genomic testing currently requires specialized labs, remains slow, and is of high cost which precludes its routine application for patients with isolated polyhydramnios, as experienced with our patient. Elevated amniotic fluid chloride levels were proposed to be of diagnostic value in some reports (13, 16), nevertheless, other studies did not support this approach as electrolytes levels were shown to be inconsistent, possibly due to a dilutional effect from the maternal extracellular fluid. Instead, the Bartter Index which is based on the multiplication of the total protein and alpha-fetoprotein (AFP) expressed as multiples of the median (MOM) was proposed as a more reliable and feasible prenatal diagnostic marker (13, 17). Other reported diagnosis clues include the presence of a consistently full fetal bladder in a case of isolated polyhydramnios (18).

Post-delivery, the differential diagnosis of BS in neonates, infants, or children, includes a wider range of disorders such as congenital chloride diarrhea (CCD), which may cause mild polyhydramnios, however the gastrointestinal tract should show some abnormal dilatation (19). In addition, a heterogeneous group of several genetic disorders often referred to as pseudo-Bartter/Gitelman syndrome overlaps ap with BS in presentation (20, 21).

Indomethacin has been proposed as an effective treatment option for antenatal BS, which acts mainly by the inhibition of prostaglandin E2 synthesis. This mechanism is crucial since elevated prostaglandin E2 in Bartter syndrome causes electrolyte abnormalities. Alternative treatment options for polyhydramnios include expectant observation or repeated amnioreduction using amniocentesis (22). Due to significant abdominal distension and pressure symptoms caused by the severe polyhydramnios, the “wait and see” approach was not tolerable by our patient. Amniocentesis has been used with other causes of polyhydramnios and with idiopathic polyhydramnios. It was also reported to be used in combination with indomethacin in a few cases of BS (23). Due to this patient's history raising a high suspicion of the diagnosis of BS, the use of indomethacin was preferred. Unlike amniocentesis, indomethacin has a therapeutic value on the fetal original condition together with its effect on the reduction of polyhydramnios and its tocolytic effect on the overly distended uterus. In our opinion, combining amniocentesis with indomethacin could have increased maternal and fetal risks such as pre-term delivery, premature rupture of membranes, fetal injuries, and maternal sepsis (24–26).

In terms of the risks associated with the antenatal use of indomethacin, several have been investigated, more commonly with the use of indomethacin for its tocolytic effect in preterm labor. The most consistently reported risk of using indomethacin is its countereffect on the patency of the fetal DA. A constricted DA can lead to pulmonary hypertension and if untreated fetal death (27). The diameter of the ductus arteriosus varies at different gestational weeks, however, generally, the mean values range from 1.34 mm at week 15–3.49 mm at week 34 (28). In the present report, the effect of indomethacin on fetal DA diameter was well-observed and rapid but reversible as seen when the dose was reduced and then when indomethacin was stopped at 32 weeks.

Other adverse effects of indomethacin include its effect on the maturation of the fetal kidneys and the total number of nephrons. In addition, there is an increase in the risk of necrotizing enterocolitis (NEC), particularly in preterm infants, and associated risk of periventricular leukomalacia (29, 30). Accordingly, there is substantial evidence that the maternal use of indomethacin during pregnancy should be very judicious. However, some studies denoted that gastrointestinal adverse effects including perforation might be of more significant clinical concern when indomethacin is used in the neonatal period (9, 31). Due to a lack of evidence on clinical sonographic markers that should be measured to predict the majority of other adverse effects of indomethacin, clinicians including the authors of this report used to focus only on the DA diameter and general fetal wellbeing measures to make decisions of when to stop the indomethacin therapy.

It was challenging to determine the optimal duration and dose of indomethacin in this patient for reducing polyhydramnios without seriously affecting the patency of the DA. Bhat et al. reviewed the data available on Batter's syndrome, suggesting that indomethacin therapy should be started as soon as the syndrome is diagnosed at a dose of 1 mg/kg/day in two divided doses, with no recommendation for the duration (2). According to Gael Abou-Ghannam et al. the initial loading dose of indomethacin was generally advised between 50 and 100 mg/day and can be increased up to 2–3 mg/kg/day (9). However, our experience in the management of this patient showed that when used at an initial dose of 2 mg/kg/day (i.e., 150 mg/day), indomethacin achieved an effective and rapid reduction in polyhydramnios within 1 week, however, it significantly reduced the diameter of the DA. Therefore, we had to reduce the dose to 50 mg/day to maintain the patency of the DA until the completion of the 32 weeks. Since indomethacin is not recommended after 32 weeks, it was suggested to be tapered down until it is stopped (9). During indomethacin therapy, the patency of the DA should be continuously monitored along with the umbilical artery PI. The decision on when to terminate the pregnancy should be based on indicators of fetal wellbeing. In our patient, indicators of fetal wellbeing including fetal movement counts, cardiotocography, biophysical profile, and Doppler studies of the umbilical artery were monitored. Severe polyhydramnios can increase the umbilical artery PI and can lead to considerable Doppler abnormalities including absent or reversed end-diastolic velocity (AREDV). Although the mechanism by which polyhydramnios affects fetal Doppler indices is not fully understood, it is suggested that the increased amniotic pressure could lead to reduced fetal cord PH and may compromise fetal cardiac function (32). The umbilical artery Doppler is a major indicator of fetal wellbeing and according to the guidelines of the Royal College of Obstetricians and Gynecologists (RCOG) on the management of small for gestational age (SGA), the delivery timing should be based on this indicator among other parameters. In an SGA fetus with umbilical artery AREDV detected before 32 weeks, delivery is recommended when either the gestational age reaches 32 weeks, when ductus venosus Doppler becomes abnormal, or when umbilical vein pulsations appear (33).

Indomethacin is known to prolong the duration of the pregnancy affected polyhydramnios (34). However, in this case, we are not confident that this effect has been significantly achieved. Despite not receiving indomethacin in her previous twin pregnancy, the pregnancy continued till 34 weeks. It is still possible that if indomethacin was not given in the second pregnancy, the severity of polyhydramnios could have led to a much earlier delivery.

Counseling mothers with fetal Bartter syndrome is another challenge. Clinicians need to provide education about this condition including expectations on pregnancy management, side effects of treatment, as well as the progressive nature of polyhydramnios and the eventual premature delivery, and the long-term prognosis of affected children.

Following delivery, women should receive adequate counseling about the specific care of the neonate and treatment options, which best be provided by a pediatric nephrologist. Ideally, multidisciplinary care should start as early as possible during pregnancy if the diagnosis is confirmed. Postnatal complications include dehydration and failure to thrive. Lifelong excessive loss of salt and water leads to growth retardation in most affected children, which may improve over time. Intellectual disability and sensorineural deafness can develop with some of its genetic variants (2, 22). Nephrocalcinosis may develop during follow-up, whereas chronic kidney disease and subsequent renal failure may complicate the condition, particularly in BS type 4 (15). Various treatment options have been proposed for newborns and children affected by BS, including evidence-based and controversial interventions as well-reviewed by Kleta et al. (35). According to recent recommendations, the mainstay of management should include high salt and water replacement, NSAIDs, potassium chloride supplements, and supportive treatment (22). Unfortunately, each treatment has its adverse effect, including the direct association between NSAIDs and the risk of NEC/gastrointestinal perforation—as has been the case with the newborn of this case study- and chronic kidney diseases (36).

In conclusion, in this case, report, we share our experience using indomethacin in the management of the antenatal Bartter syndrome using indomethacin. We discuss the optimization of dose and duration of therapy using a combination of AFI, DA diameter, and umbilical artery Doppler indices as clinical indicators to guide the effectiveness, duration, and safety of therapy and delivery timing.

## Data Availability Statement

The original contributions presented in the study are included in the article/supplementary material, further inquiries can be directed to the corresponding author/s.

## Ethics Statement

Ethical review and approval was not required for the study on human participants in accordance with the local legislation and institutional requirements. Written informed consent to participate in this study was provided by the participants' legal guardian/next of kin. Written informed consent was obtained from the individual(s), and minor(s)' legal guardian/next of kin, for the publication of any potentially identifiable images or data included in this article.

## Author Contributions

OA wrote the first draft of the manuscript and contributed to the scientific literature search. MS contributed to initial data collection, performed the scientific literature search, and contributed to manuscript writing. GM edited and contributed to the revision of the manuscript. UH contributed to data collection and reviewed the final version of the manuscript. NM guided all editing and reviewed the manuscript. All authors reviewed and agreed on the final version of this manuscript.

## Conflict of Interest

The authors declare that the research was conducted in the absence of any commercial or financial relationships that could be construed as a potential conflict of interest.

## Publisher's Note

All claims expressed in this article are solely those of the authors and do not necessarily represent those of their affiliated organizations, or those of the publisher, the editors and the reviewers. Any product that may be evaluated in this article, or claim that may be made by its manufacturer, is not guaranteed or endorsed by the publisher.
